# Supplementary material for: A geospatial source selector for federated GeoSPARQL querying
Source: Open Res Eur. 2022 Oct 6;2:48. Originally published 2022 Apr 19. [Version 2] doi: 10.12688/openreseurope.14605.2 (PMC10446020; doi:10.12688/openreseurope.14605.2)
Supplement: Peer review history

# Peer review: Reviewer response for version 2

**DOI:** 10.21956/openreseurope.16399.r30243

**Published:** 2022-10-21

**Authors:** Markus Stocker

**Affiliations:** 1 TIB - Leibniz Information Centre for Science and Technology, Hannover, Germany

The authors have addressed reviewer comments reasonably well. I think we can debate on the impact of the work and the practical relevance of the technologies for much longer, so perhaps this discussion is material for another paper.

Is the rationale for developing the new method (or application) clearly explained?

Partly

Is the description of the method technically sound?

Yes

Are the conclusions about the method and its performance adequately supported by the findings presented in the article?

Yes

If any results are presented, are all the source data underlying the results available to ensure full reproducibility?

Partly

Are sufficient details provided to allow replication of the method development and its use by others?

Yes

Reviewer Expertise:

My areas of expertise include semantic web technologies as well as geospatial data and query processing.

I confirm that I have read this submission and believe that I have an appropriate level of expertise to confirm that it is of an acceptable scientific standard.

# Peer review: Reviewer response for version 2

**DOI:** 10.21956/openreseurope.16399.r30242

**Published:** 2022-10-20

**Authors:** Ozgu Can

**Affiliations:** 1 Department of Computer Engineering, Faculty of Engineering, Ege University, Bornova, Turkey

My comments have been mostly resolved. I suggest authors to add the following issues regarding the current version of the manuscript before the final publication:

- Figure 4 is very superficial to demonstrate the architecture. The figure could be improved.
- Authors claim that real-life use cases are limited. Thus, the limitations of the study should be discussed in the conclusion.

Is the rationale for developing the new method (or application) clearly explained?

Partly

Is the description of the method technically sound?

Partly

Are the conclusions about the method and its performance adequately supported by the findings presented in the article?

Yes

If any results are presented, are all the source data underlying the results available to ensure full reproducibility?

Yes

Are sufficient details provided to allow replication of the method development and its use by others?

Yes

Reviewer Expertise:

Semantic Web, Knowledge Management, Ontology Engineering, Security, Privacy,

I confirm that I have read this submission and believe that I have an appropriate level of expertise to confirm that it is of an acceptable scientific standard.

# Peer review: Reviewer response for version 1

**DOI:** 10.21956/openreseurope.15771.r29516

**Published:** 2022-07-20

**Authors:** Markus Stocker

**Affiliations:** 1 TIB - Leibniz Information Centre for Science and Technology, Hannover, Germany

The authors propose an approach that enables GeoSPARQL queries to determine which sources to geospatial data to query, in a federated setting. The work is technically sound and evaluated sufficiently well. However, I personally found it very hard to read. Aside from occasional typos, sentences are ofter far too long and complicated. I think authors should substantially revise the manuscript and improve readability.

 The authors claim that federated query processing is urgently needed to achieve "full" integration of geospatial data in the Semantic Web. Not sure I agree, since spatial querying in the Semantic Web is not obvious even on standalone databases. More generally, how much of a problem is it actually? The Semantic Web has been loosing ground for some time; the adoption of RDF and GeoSPARQL in (research) infrastructure is rare and the prospects not exactly great (in my opinion). On the other hand, there is solid GIS tech out there (PostGIS, JTS Topology Suite, etc.) that do an excellent job and seem to do just fine without RDF, GeoSPARQL, etc. Hence, while I appreciate the academic exercise of developing source selectors for GeoSPARQL I do wonder how much of a problem it actually is.

 For an academic work, I am afraid it does not cover well enough the literature. The authors limit the review to the Semantic Web area, which is not adequate because more related work has been done outside the Semantic Web area. Moreover, foundational work such as RCC are not reviewed adequately. Even within the Semantic Web area, there is related work that has not been considered. My suggestion is that the manuscript should have 2-3 times as many citations.

 In Definition 2, the authors suggest that the domain of the newly introduced property is void:Dataset. What is the implication of this restriction? Does the approach only work for individuals typed void:Dataset. What about all the other applicable types, say, qb:DataSet or dcat:Dataset? The choice seems to be very restrictive.

 Most of the results are obvious, expected and thus unsurprising. For instance, it does not surprise that the thematic source selector is faster than geospatial source selectors, since it is computationally much cheaper to match some URIs than to perform polygon computations. The same can be said about source selection pruning: Of course the selector does a better job the more precise the information available.

 The overall conclusion seems that "difficult queries" rely on precise bounding polygons while less accurate descriptions are sufficient for "simpler queries". As the authors phrase it: "no size fits all." What does this mean in practice? Do all datasets have to be described with both precise and less-precise bounding boxes, so that the system can choose based on query complexity? And if so how would this concretely work? What is the recommendation?

 As a minor remark, I suggest to move the result tables closer to the corresponding text. It was rather difficult to switch back and forth between text and tables.

Is the rationale for developing the new method (or application) clearly explained?

Partly

Is the description of the method technically sound?

Yes

Are the conclusions about the method and its performance adequately supported by the findings presented in the article?

Yes

If any results are presented, are all the source data underlying the results available to ensure full reproducibility?

Partly

Are sufficient details provided to allow replication of the method development and its use by others?

Yes

Reviewer Expertise:

My areas of expertise include semantic web technologies as well as geospatial data and query processing.

I confirm that I have read this submission and believe that I have an appropriate level of expertise to confirm that it is of an acceptable scientific standard, however I have significant reservations, as outlined above.

# Response

**Published:** 2022-09-29

**Authors:** Antonis Troumpoukis

**Affiliations:** National Center for Scientific Research (NCSR) Demokritos, Greece

Regarding the comment on the (lack of) adoption of geospatial RDF/SW technologies, and on GeoSPARQL losing ground, we would like to react by pointing out that GeoSPARQL is an OGC standard; That goes to show that, although W3C was also naturally involved, the main drive for developing the standard comes from the geospatial databases community. GeoSPARQL has also been implemented by Oracle, with the relevant reference added in the article. In any case, we would also like to point out that the work described here is shown to lead to greater efficiency, thus creating an opportunity for greater adoption of linked geospatial data in general and of federated linked geospatial data in particular. This would allow GIS technologies (including mature systems such as Oracle, with a demonstrated interest in GeoSPARQL) to also participate in the decentralization drive of the modern Web. We have rephrased the conclusions to more clearly spell out that we need to complement the work described here with work on predicting the run-time complexity of queries. This is clearly future work that relates to the query optimizer (rather than the source selector), so we give some initial thoughts in this direction in the final section. We have expanded the related work section with more discussion on relevant literature and how our work is positioned with respect to it. We have also expanded the background, including adding a reference to RCC8. Regarding the relationship between void:Dataset, qb:Dataset, and dcat:Dataset we feel that void:Dataset is the most appropriate term since it aims at annotating a SPARQL endpoint with useful statistics and summaries. qb:Dataset is too restrictive, since it also prescribes internal structure. dcat:Dataset is too generic, as it is not restricted to SPARQL endpoints but can be used for any data that is available for downloading. We feel that adding such minute SW details to this article would be a regression, but we can add it if you insist with your comment. We do not understand why the data underlying the results are commented as being partly available. All data and software needed to reproduce the experiments is published online. Regarding the positioning of the figures and tables, I am afraid that this is done by the journal’s editors, outside our control. We have fixed several typos and broken long sentences into shorter, more readable ones.

# Peer review: Reviewer response for version 1

**DOI:** 10.21956/openreseurope.15771.r29515

**Published:** 2022-06-30

**Authors:** Ozgu Can

**Affiliations:** 1 Department of Computer Engineering, Faculty of Engineering, Ege University, Bornova, Turkey

The manuscript proposes a geospatial source selector for geospatial data, presents the implementation of the related source selector and the correctness of the proposed source selection. Also, evaluation results are compared.

 The manuscript should address the following issues before indexing:

 The motivation is incomplete. In the Introduction, the problems that the manuscript is focused on should be expressed in more detail with solid examples. Moreover, the state of the art should be discussed in more detail. The unique contribution needs to be clarified. For this purpose, contributions should be listed in a subsection. The main problems of federated query processing should also be presented and discussed. Also, the background section could be improved (such as with federation approaches, etc). Real-life use cases should be given and the proposed study should be discussed by considering the widespread effect of the proposed source selection. The literature work should be elaborated more. Also, recent studies in the field should be added and the review of the related works should be detailed. Further, some illustrations could be added to strengthen the presented manuscript, such as an example of a GeoSPARQL query, the overall architecture of the query processing and source selection, etc. The organization and readability of the manuscript needs to be improved.

Is the rationale for developing the new method (or application) clearly explained?

Partly

Is the description of the method technically sound?

Partly

Are the conclusions about the method and its performance adequately supported by the findings presented in the article?

Yes

If any results are presented, are all the source data underlying the results available to ensure full reproducibility?

Yes

Are sufficient details provided to allow replication of the method development and its use by others?

Yes

Reviewer Expertise:

Semantic Web, Knowledge Management, Ontology Engineering, Security, Privacy,

I confirm that I have read this submission and believe that I have an appropriate level of expertise to confirm that it is of an acceptable scientific standard, however I have significant reservations, as outlined above.

# Response

**Published:** 2022-09-29

**Authors:** Antonis Troumpoukis

**Affiliations:** National Center for Scientific Research (NCSR) Demokritos, Greece

We have added an explicit list of contributions in the “Introduction” section. We have added material that motivates our work in the “Motivation and use case” section. We have expanded the related work section with more discussion on relevant literature and how our work is positioned with respect to it. We have also expanded the background with a more elaborated presentation of federated query processing systems (including architecture diagram). Finally, we have added an illustrative example of a GeoSPARQL query in the main methodological section. Real-life use cases are limited by the lack of technical infrastructure to implement them, infrastructure which is what we are presenting in this article. But, as we note in the article, it is a real-world phenomenon that datasets are partitioned either by jurisdiction or by gridding. This motivates our experimental setup, where we have successfully federated unaligned layers where one layer is partitioned by jurisdiction and another by gridding.
